# Addenbrooke's Hospital

**Published:** 1900-02-10

**Authors:** 


### The Institutional Workshop.

### ADDENBROOKE'S HOSPITAL.

It will be within the recollection of our readers that in consequence of the financial and other difficulties by which Addenbrooke's Hospital, Cambridge, found itself beset, a couple of years ago, the Governors asked Sir Henry Burdett to investigate the general condition of the charity, and to advise them as to what should be done to place it upon a satisfactory footing. After giving considerable attention to the matter, and investigating the position of the institution in regard to its constitution, expenditure, and income; conferring with the governing body; and careful inspection of the building and its arrangements, Sir Henry Burdett, in June, 1898, presented his report to the Special Finance Committee of the Governors.

In this report he recommended that a Selection Committee for the purpose of appointing the Honorary Medical Officers should be set up at the first General Court in each year, and that it should consist of 50 Governors, 18 of whom should be members of the Committee of Management, 8 Honorary Physicians and Surgeons for the time being, and 24 other Governors, namely, eight from each of the three groups of Governors recognised in the Act of Parliament constituting the hospital, namely, the County, the Borough, and the University; that a new bye-law should be passed prohibiting canvassing on the part of candidates; that in lieu of the open Weekly Board of Governors, at which any Governor who pleased was able to attend and vote, a Committee of Management should be appointed, consisting of a certain number of Governors, with some or all of the Medical Staff, and that one-third of this Committee should retire every year, but be eligible for re-election; that out of this Committee an Executive Committee and a Finance Committee should be appointed; and that an active Governor should be appointed as Chairman of the Committee of Management, who should have power to decide matters of importance arising between the meetings of the Committee.

In regard to economy and finance Sir Henry Burdett made many important recommendations, among which was one to the effect that all legacies as received should be placed to deposit account at the bankers', at interest, and that the investment of such legacies or the sale of stock should be settled at the end of each financial year by the Committee of Management, on the recommendation of the Treasurer and Finance Committee; and, finally, among a series of general recommendations, there was one in favour of the appointment of a young, experienced, and active man as resident superintendent.

This report, although approved of by the committee to which it was presented, was not acted on by the Board, which, being an open board, was practically at the mercy of any obstructive old gentlemen who chose to attend. But in May, 1899, another Committee of Governors was appointed, who have recently brought up another report, embodying some of the proposals made by Sir Henry Burdett—though without any mention of his name—and also introducing important arrangements with the University in regard to the position of the Professors of Medicine and of Surgery on the Staff, the teaching of the students, and the conduct of the examinations in the hospital; and the recommendations of this second committee have now been presented to a Special Court of Governors, and have been adopted.

The reforms now adopted include the following points: (*a*) That all tenders should be advertised for and should be open, and that the contracts should, as a rule, be yearly. (*b*) That in order to conduct and maintain an organised system of collection of money from various sources, to look after the repair of the hospital and its outbuildings, to superintend the delivery and check the consumption of stores, to examine bills, &c., and generally to superintend all matters outside the duties of the Medical Staff and the Matron, a competent retired naval or military officer be appointed as superintendent. It is added, however, that he need not reside in the hospital, an arrangement which, we fear, will detract greatly from his usefulness. (*c*) That an arrangement with the University be made, in accordance with which the Staff will ultimately relinquish to the hospital two-eighths of the fees received from the students. (*d*) That certain other arrangements be made in regard to the teaching and examination of the students, the most important of which is to the effect that the present grant of £50 per annum from the University to the Hospital shall be raised to £300 per annum, on condition that "the Governors, on the application in writing of the Vice-Chancellor, elect the Regius Professor of Physic an Honorary Physician, and the Professor of Surgery (if any) an Honorary Surgeon of the Hospital; such respective Professor to hold office during the tenure of his Professorship," and "that the Governors, on the application in writing of such Physician or Surgeon respectively, assign to him a share, to the extent of one-fourth, of the beds in his respective department." Neither of the said Professors are to receive any share of the fees paid by the students to the Medical Staff, which accounts for the "two-eighths" of the fees to be relinquished by the Staff to the hospital mentioned above. (*e*) That an Advisory Council be formed consisting of fifteen Governors, who shall take over the duties of the Finance Committee, examine the testimonials of candidates for vacancies in the Honorary Staff, and report to the Governors which candidate they consider best qualified to fill the appointment; and that in the formation of this Advisory Council the town, the county, and the University shall be represented in equal proportions.

In the conduct of the negotiations which have led to the adoption of this report, and the acceptance of these reforms, much credit is due to Dr. MacAlister and Mr. Finch. It is clear, however, that the whole working and organisation of the charity could be greatly simplified if some of the restrictions imposed by the Act constituting the hospital could be got rid of. The Lord Lieutenant of the County, Mr. Peckover, has proved himself a munificent benefactor to Addenbrooke's, and he could render it one more service if he would undertake the cost of obtaining an amended Act of Parliament, whereby the constitution of the hospital could be so modernised that Addenbrooke's should fill its proper place, not merely as the local hospital for the town and county—although this position would still be filled even better than it has hitherto been done—but also as an integral part of what is rapidly becoming the most important medical school in England.

